# Mixed Infection (Meningococcus and Pneumococcus) Meningitis

**Published:** 1919-01

**Authors:** 


***Mixed Infection (Meningococcus and Pneumococcus) Meningitis.*** By J. G. FITZGERALD, *Journal of the American Medical Association.* Sept. 21, 1918.

The author records a series of reports on mixed meningococcus and pneumococcus infection made by Netter, Salanier, and Mathers. All cases observed were in children and the primary infecting micro-organism was the meningococcus. Although the frequency of such infection has not yet been determined, it is believed to occur in about 5 % of the cases, possibly in more. Thus, in a series of twelve cases of meningitis, both the meningococcus and pneumococcus were found in three.

The author points out the desirability of recording the presence of the pneumococcus in the cerebrospinal fluid. He also states that in the study of cerebrospinal meningitis clinical and laboratory observations should be made in closer correlation ; laboratory examination of a few samples of cerebrospinal fluid is not sufficient. If a record of the number of cases of pure meningococcus meningitis subsequently showing the presence of pneumococcus in the cerebrospinal fluid is to be drawn up, all samples of the fluid withdrawn must be examined bacteriologically.

Such lack of correlation between clinical and laboratory observation, and the highly probable fact that not all the observers of meningococcus meningitis were able to submit to bacteriologic examination every specimen of the cerebrospinal fluid withdrawn, leaves the existing statistics as to the value of anti-meningitis serum, in the treatment of epidemic meningococcus meningitis, open to criticism; it has also hampered the work of the Meningitis Division of the Research Laboratory of the Health Department of New York City. Unless every sample of the cerebrospinal fluid withdrawn is examined bacteriologically, no precise opinion can be formed as to the effect of serum treatment.

In many hospitals, both in America and Europe, the bacteriologic and clinical studies of cases of meningococcus meningitis have been carefully correlated, and the importance given to the treatment and study of cerebrospinal meningitis in the army has formed the basis for further efforts in that direction. A special organization has been established in England for this purpose under the Medical Research Committee.

In a series of 12 cases of cerebrospinal meningitis seen in a military base hospital in Canada, the infection in 9 cases was due to the meningococcus alone, and in all these the patients recovered. The 3 remaining cases were as follows :

In case 1, only the pneumococcus was found in culture; but as the patient died very soon after admission to hospital, complete cultural studies were not possible, and this case cannot be considered as a sure case of mixed pneumococcus and meningococcus infection.

In case 2, the patient's condition improved so after 4 to 5 doses of serum that recovery was expected, although the bacteriologic examination gave indications as to the probability of a fatal outcome some time before any marked change occurred in the patient's general condition. The pneumococcus isolated was a strain of type 1.

In case 3, there was a marked improvement in the patient's condition, followed as in case 2 by a swift change and death. The final film preparations showed a great number of gram-positive diplococci, which on culture proved to be pneumococci, type 2.

With the exception of 3 cases reported by Netter and Salanier, none of the patients suffering from mixed meningococcus and pneumococcus infection recovered.

When the organism producing the mixed infection is a strain of pneumococcus type 1, type 1 anti-pneumococcus serum should be used.

